# Retrospective study of the differences in patient characteristics and revenue between male and female surgeons in Taiwan

**DOI:** 10.1038/s41598-021-03289-6

**Published:** 2021-12-09

**Authors:** Weiming Cheng, Shu-Yi Lin, Yu-Hua Fan, Sheng-Wen Chen

**Affiliations:** 1 Division of Urology, Department of Surgery, Taipei City Hospital, Zhongxiao Branch, Taipei, Taiwan; 2 Department of Education and Research, Taipei City Hospital, Taipei, Taiwan; 3 Department of Urology, Taipei Veterans General Hospital, Taipei, Taiwan; 4 Program in Molecular Medicine, School of Life Sciences, National Yang Ming Chiao Tung University, Taipei, Taiwan; 5 Institute of Biopharmaceutical Science, School of Life Science, National Yang Ming Chiao Tung University, Taipei, Taiwan; 6 Department of Urology, Faculty of Medicine, National Yang Ming Chiao Tung University, Taipei, Taiwan; 7 Urological Center, No.87, Tongde Road, Nangang District, Taipei, 11556 Taiwan

**Keywords:** Health care, Health occupations, Urology

## Abstract

Surgery is traditionally a male-dominated field, and gender differences exist despite the growing numbers of female surgeons. A handful of studies have evaluated the condition in Asian societies. We aimed to examine the difference between female and male surgeons in urology, general surgery, and gynecology by analyzing a nationwide, population-based database. We identified surgeons with a clinical experience of six to thirteen years between 1995 to 2013 from the National Health Insurance Research Database. We collected patient numbers and revenue per month in outpatient and inpatient care, as well as monthly numbers of surgeries conducted by female and male surgeons in urology, general surgery, and gynecology, for analysis. Original student’s t-test and wilcoxon rank sum test was used to compare the differences between female and male surgeons, and p values less than 0.05 were considered statistically significant. Female urologists and general surgeons had a significantly higher ratio of female patients in Taiwan. Female urologists had patient numbers, revenues, and numbers of surgeries comparable to male urologists. In contrast, female general surgeons had significantly less involvement in outpatient and inpatient care and had low monthly revenues. Female general surgeons contradictorily performed more oncological surgeries per month than males. However, the difference in numbers of oncological surgeries was not significant after excluding breast cancer surgeries. Female gynecologists had a similar amount of outpatients and outpatient revenue but significantly less inpatient care and numbers of surgeries per month. A gender-based gap exists among surgeons in Taiwan. The gap between females and males appeared narrower in urology than in general surgery and gynecology. Management of diseases related to female sex organs, including breast, were more common among female surgeons. Efforts should be made to decrease gender stereotypes, to ensure that patients receive the best care regardless of the sex of the surgeons.

## Introduction

A significantly large number of females have entered the field of medicine in the past few decades. In Taiwan, 36.6% of total new medical graduates were female in 2018. Taiwan Medical Association estimates that female physicians compose nearly 20% of the total healthcare workforces^1^. As the growing population in the field of medicine, female physicians have their advantages and limitations in clinical practices. For example, female physicians tend to have thorough and empathetic communication patterns that make patients feel understood and improve doctor-patient relationships^2,3^. However, sex segregation still presents^4^ and influences the choice of specialties as their career for females physicians. A previous study has pointed out that female physicians were less likely to specialize in surgery than males^5^, and the gap in practice pattern and salaries have also been documented by some studies^6–11^. Nevertheless, most studies evaluated surgeons in America and Europe, where cultures are different from that in Asia. There is a lack of studies concerning the gender gap in surgery in Asian societies, which are traditionally viewed as more conservative. We assessed the gender gap among surgeons by analyzing a nationwide, population-based database in Taiwan and focused on three sub-specialties that included the management of disease of female sex organs: urology, general surgery, and gynecology.

## Materials and methods

### Data source

We queried the National Health Insurance Research Database (NHIRD), containing registration files and medical data of approximately 23 million Taiwanese residents (98% of the population). All data from NHIRD are anonymous and scrambled for privacy protection. Researchers who request the use of NHIRD must sign a written agreement to declare that they will comply with the privacy protection rules for patients and care providers. We also examined Longitudinal Health Insurance Dataset 2000 (LHID 2000), a sub-dataset of NHIRD, including all the medical insurance information from one million randomly selected residents in the year 2000.

### Study population

Certified urologists, gynecologists, and general surgeons who practiced medicine for six to thirteen years between 1995 to 2013 were identified. Surgeons with clinical practice over thirteen years were excluded from our research since senior surgeons might reduce case complexity and develop specialized practices^12^. In contrast, young surgeons whose clinical experience was less than six years were also excluded due to irregularities in establishing patient base and reputation.

The monthly number of patients and their sex ratio, the total numbers of surgeries conducted, and the revenue from outpatient and inpatient care by each surgeons were obtained from the database. We did not present data on the sex distribution of patients in the gynecology practice since almost all of these patients were female.

The numbers of oncological surgeries, which required longer operation time and higher physical strength, was sub-analyzed. The oncological surgeries were defined as: (1) radical surgeries for prostate cancer, urothelial carcinoma of the urinary tract, kidney cancer, penile cancer, testicular cancer, and retroperitoneal tumor in urology; (2) radical surgeries for breast cancer, lung cancer, esophageal cancer, gastric cancer, colon cancer, liver cancer, and pancreatic cancer in general surgery; and (3) radical surgeries for vulvar cancer, vaginal cancer, uterus and cervical cancer, and ovarian cancer in gynecology.

For oncological surgeries in general surgery, we analyzed total numbers of oncological surgeries as well as total numbers of oncological surgeries excluding breast cancer because female patients may prefer female surgeons when it comes to sex organ-related disorders. Moreover, radical surgery for breast cancer is not as time-consuming as other oncological surgeries in general surgery, and breast cancer surgery is reportedly the most frequent primary procedure performed by female general surgeons^9^.

Since both urologists and gynecologists performed transvaginal surgeries, including surgeries for urinary incontinence, pelvic organ prolapse, and vaginal fistula, we also sub-grouped these surgeries for evaluation.

### Statistical analysis

Since the population of female surgeons included in the study was small, Kolmogorov–Smirnov test and Shapiro–Wilk test were firstly performed to examinate whether the distribution was normal or not. Kolmogorov–Smirnov test showed that normal distribution was reached in general surgeon and gynecologist groups, respectively. Hence, original student’s t-test was then used to test the differences of continuous variables between male and female general surgeons as well as gynecologists. On the other hand, neither Kolmogorov–Smirnov test nor Shapiro–Wilk test proved that normal distribution was reached in urologist group. Nonparametric methods were suitable for statistical analysis in urologist group. Wilcoxon rank sum test was performed for the comparison between the male and female urologists. All statistical analyses were performed using IBM SPSS Statistics for Windows, ver. 24 (IBM Corp., Armonk, NY, USA). A p-value of less than 0.05 was considered statistically significant.

## Results

A total of 13, 87, and 191 female urologists, general surgeons, and gynecologists, respectively, were included in this study, accounting for 6.7%, 7.0%, and 51.3%, respectively, of surgeons in each specialty. The differences in numbers of patient care and revenue as well as numbers of surgeries performed between female and male surgeons in urology, general surgery, and gynecology are listed in Tables 1, 2, and 3.

**Table 1:** Comparison of numbers of patients, surgeries, revenue, and sex ratio of patients treated by female and male urologists.

	Female urologists	Male urologists	p value
	N = 13	N = 180	
	Median	Median	
**Patient numbers per month**			
Outpatients	111.33	138.86	0.285
Male to female sex ratio	0.60	0.74	**< 0.001**
Inpatients	2.50	4.26	0.213
Male to female sex ratio	0.58	0.74	**0.005**
**Total revenue per month, NTD**	283,861.80	401,504.92	0.077
From outpatient care, NTD	173,131.63	230,487.87	0.103
From inpatient care, NTD	81,650.17	149,602.69	0.166
**Total number of surgeries conducted per month**	2.13	3.54	0.278
Oncological surgery	0	0	0.718
Transvaginal surgery	0	0	0.095

Significant values are in bold.

*NTD* New Taiwan Dollar.

**Table 2:** Comparison of numbers of patients, surgeries, revenue, and sex ratio of patients treated by female and male general surgeons.

	Female surgeons		Male surgeons		p value
	N = 87		N = 1157		
	Mean	SD	Mean	SD	
**Patient numbers per month**					
Outpatients	66.02	57.11	94.77	98.93	**< 0.001**
Male to female sex ratio	0.47	0.19	0.56	0.13	**< 0.001**
Inpatients	2.73	2.71	3.98	3.86	**< 0.001**
Male to female sex ratio	0.53	0.25	0.61	0.18	**0.010**
**Total revenue per month, NTD**	276,178.19	259,484.63	425,444.54	369,009.56	**< 0.001**
From outpatient care, NTD	110,827.09	109,540.88	139,242.61	128,728.28	**0.045**
From inpatient care, NTD	165,351.10	196,125.27	286,201.93	312,943.54	**< 0.001**
**Total number of surgeries conducted per month**	2.30	2.50	3.28	3.33	**0.001**
Oncological surgery	0.33	0.64	0.17	0.41	**0.030**
Oncological surgery excluding breast	0.07	0.22	0.12	0.32	0.057

Significant values are in bold.

*NTD* New Taiwan Dollar.

**Table 3:** Comparison of numbers of patients, surgeries, revenue, and sex ratio of patients treated by female and male gynecologists.

	Female gynecologist		Male gynecologist		p value
	N = 191		N = 181		
	Mean	SD	Mean	SD	
**Patient numbers per month**					
Outpatients	174.94	150.84	173.94	143.71	0.948
Inpatients	1.94	2.79	4.72	4.85	**< 0.001**
**Total revenue per month, NTD**	171,195.02	137,490.23	277,742.18	237,122.05	**< 0.001**
From outpatient care, NTD	99,825.67	74,559.70	110,258.87	123,435.43	0.328
From inpatient care, NTD	71,369.34	98,667.67	167,483.31	173,151.48	**< 0.001**
**Total number of surgeries conducted per month**	1.76	2.62	4.37	4.60	**< 0.001**
Oncological surgery	0.04	0.15	0.12	0.28	**0.002**
Transvaginal surgery	0.03	0.12	0.08	0.23	**0.005**

Significant values are in bold.

*NTD* New Taiwan Dollar

### Sex ratio of patients

The ratio of female outpatients and inpatients treated by female urologists was significantly higher than those treated by male urologists (male-to-female patient ratio 3.39 ± 3.07 versus 4.83 ± 3.92, p = 0.004; 0.58 ± 0.21 versus 0.73 ± 0.14, p = 0.030, respectively). Similar conditions were observed for female general surgeons (male-to-female patient ratio 0.47 ± 0.19 versus 0.56 ± 0.13, p < 0.001 in outpatient care; 0.53 ± 0.25 versus 0.61 ± 0.18, p = 0.010 in inpatient care).

### Monthly patient numbers and revenue

The monthly patient numbers and revenue generation in outpatient and inpatient care by female urologists were non-inferior to those by male urologists (Table 1); however, female general surgeons had significantly fewer numbers of outpatients and inpatients per month (66.02 ± 57.11 versus 94.77 ± 98.93, p < 0.001; 2.73 ± 2.71 versus 3.98 ± 3.86, p < 0.001, respectively). The total revenue per month of female general surgeons was significantly lesser than that of male general surgeons (NTD$276,178.19 ± 259,484.63 versus NTD$425,444.54 ± 369,009.56, p < 0.001; Table 2). The number of outpatients treated by female gynecologists was comparable to that by male gynecologists (174.94 ± 150.84 versus 173.94 ± 143.71, p = 0.948); however, the monthly inpatient numbers were less (1.94 ± 2.79 versus 4.72 ± 4.85, p < 0.001). Revenue contribution from outpatient care by female gynecologists was comparable to that of male gynecologists (p = 0.328); however, the revenue of inpatient care by female gynecologists was significantly lesser (NTD$71,369.34 ± 98,667.67 versus NTD$167,483.31 ± 173,151.48, p < 0.001). The total monthly revenue of female gynecologists was also significantly lesser (NTD$171,195.02 ± 137,490.23 versus NTD$277,742.18 ± 237,122.05, p < 0.001; Table 3).

### Numbers of surgeries conducted

Regardless of their sex, urologists in Taiwan performed similar number of surgeries per month (3.01 ± 2.78 versus 4.17 ± 3.50, p = 0.245). No differences in the number of oncological surgeries and transvaginal surgeries were seen between female and male urologists (Table 1). Although female general surgeons performed fewer surgeries per month (2.30 ± 2.50 versus 3.28 ± 3.33, p = 0.001), they performed more oncological surgeries monthly (0.33 ± 0.64 versus 0.17 ± 0.41, p = 0.030). If we excluded the radical operations of breast cancer, which were performed mostly by female general surgeons, the number of oncological surgeries performed by male general surgeons was higher than that performed by female general surgeons; however, this was not statistically significant (0.07 ± 0.22 vs. 0.12 ± 0.32, p = 0.057) (Table 2). On the other hand, female gynecologists had significantly fewer surgeries per month (1.76 ± 2.62 versus 4.37 ± 4.60, p < 0.001), fewer oncological surgeries (0.04 ± 0.15 versus 0.12 ± 0.28, p = 0.002) and fewer transvaginal surgeries (0.03 ± 0.12 versus 0.08 ± 0.23, p = 0.005) than male gynecologists (Table 3).

## Discussion

This study showed that female urologists and general surgeons treated significantly more female patients. Although female urologists had comparable patient numbers, revenues, and numbers of surgeries per month as their male counterparts, female general surgeons provided significantly lesser outpatient and inpatient care and had lesser monthly revenues. Although female general surgeons performed more oncological surgeries per month than their male colleagues, there was no significant difference in numbers of oncological surgeries after exclusion of breast cancer surgeries. We also observed that female gynecologists had comparable numbers of outpatients and revenue from outpatient care but significantly lesser monthly numbers of inpatients and numbers of surgeries. To the best of our knowledge, this is the first nationwide study in Asia to examine the gender gap in the field of surgery. Exceptionally, gender gaps in numbers of patients, surgeries, and revenue were not evident in urology. Additionally, female gynecologists did not have advantages over their male counterparts in what concerns numbers of inpatients, surgeries, and revenue. These trends vary from those prevalent in western literatures^13–15^. Gender gap is likely multifactorial and warrants further exploration.

Despite the narrowing sex ratio of medical students in the past decades, gender disparity remains in certain medical specialties. Female physicians, as a growing population in the physical workforce, have greater opportunity to devote themselves to the field of pediatrics, family medicine, internal medicine, and obstetrics-gynecology^4,16,17^. Surgery is pervasively perceived as a male-dominant field. In the present study, less than 10% of urologists and general surgeons were female in Taiwan. There are several obstacles, including sexual discrimination, paucity of female role models, and work-life imbalance, for female physicians to choose surgery as their career^11,18–20^. Sexual discrimination from the patients, trainers, or colleagues is the most bothering issue to female surgeons. For example, they are frequently labeled as nursing staff or refused by male patients owing to embarrassment^20,21^. Barnes et al*.* also reported that female surgical trainees in male-dominant fields have more microaggression experiences than those in female-dominant fields^11^. Moreover, female trainees have been reported to have less autotomy granted by faculty than male trainees of the same levels in the operation room^8^.

Sex disparity in wages in surgical subspecialties had been well described in several studies, which might be an obstacle for female surgeons to develop their career^6,7,9,22,23^. We found that female general surgeons and gynecologists generated significantly less revenue than male general surgeons. Although we were unable to get the exact salaries of female and male surgeons directly from LHID2000, the revenue from diagnosing and treating patients could reflect the differences in the incomes between female and male surgeons. The cause of sex-based wage gap is multifactorial. For example, the marital status and practices pattern of female and male physicians would contribute to the wage gap. Okoshi et al. reported that the annual income of male physicians increased with an increase in the number of children, while that of female physicians decreased^9^. In the present study, female gynecologists tended to provide outpatient care more frequently, while male gynecologists care inpatients more frequently, which might result in disparities in their wages.

Both female general surgeons and gynecologists performed fewer surgeries than male general surgeons and gynecologists. This may indicate that gender stereotype may have a negative influence on female surgeons in certain aspects. Sharoky et al*.* have proved that female and male surgeons with similar background could achieve equivalent postoperative outcome when treating similar patients^24^. An online survey by Ashton-James et al. showed that male surgeons received higher ratings for their knowledge, skill, and capability from patients, while female surgeons scored higher in goodwill, empathy, and beneficence^25^. Patients would choose male rather than female surgeons when they needed surgeries, especially major oncological surgeries. Furthermore, female surgeons would voluntarily change their practice patterns, which would affect their numbers of patients and surgeries directly. In our present study, we found that despite a comparable amount of outpatients, female gynecologists had significantly less inpatients and surgeries. As observed by Antonoff and Brown, to be a wife and a mother, even a single woman, female surgeons must modify their practice pattern to achieve work-life balance^26^.

On the other hand, gender stereotype may contradictorily exert a positive impact on female surgeons to some extent, especially when history taking, physical examinations, and surgical procedures involve the female sex organs^27^. No gender preference was observed in other surgical subspecialties not involving sex organs, such as orthopedics or plastic surgery^28,29^. In the present study, female and male urologists had comparable performance regarding numbers of patients, surgeries, and revenue. This finding is compatible with those of other studies concerning urological patients^30,31^. Similarly, we found that female general surgeons performed more radical breast cancer surgeries in Taiwan. A Greek study showed that about half of women who were previously exposed to female surgeons preferred female breast surgeons^13^. Patients’ feeling of being understood, less embarrassed, and less anxious, and previous positive experience with same-sex surgeons are major advantages of female urologists and breast cancer surgeons in clinical practice.

Interestingly, Nam et al*.* reported that female urologists in the United States were favored to deliver female-specific care. However, the compensation derived from the care of oncological patients was significantly lower for female urologists compared to male urologists^7^. Female urologists in Taiwan performed a similar volume of transvaginal and oncological surgeries compared to their male counterparts. Oberlin et al. reported that among every subspecialty, female urologists operated on a greater proportion of female patients than their male colleagues^32^. This might account for the comparable performance between male and female urologists in Taiwan. Despite the challenges for females to become surgeons, becoming urologist may be a good choice for females in the Asian culture. Lifestyle, diversity of procedures, and combination of the practice of medicine and surgery might be the most positive influential factors for female physicians to pursue urology^33–35^.

There are several limitations of the present study. First, LHID 2000 did not include information on the subspecialties of individual surgeons. Therefore, we included surgeons with 6–13 years of experience; this range ensured that the surgeons were well-trained and not subspecialty-focused. Second, the information on surgeons who did not join the National Health Insurance despite being few in number was missed in the LHID 2000. Third, the revenue contribution from diagnosing and treating patients would be underestimated because data on the self-pay service could not be obtained from the LHID 2000. Fourth, we did not use questionnaires to gather information on marital status of surgeons, as well as their subjective perceptions, motivation factors, and struggles.

## Conclusion

Female urologists and male urologists had a comparable career in terms of patient numbers, revenue, and numbers of surgeries in Taiwan. Except for oncological breast surgery, female general surgeons performed less well than their male counterparts. Female gynecologists did not have any advantages over male gynecologists. Management of diseases concerning female sex organs, including the breasts, should be a preferred choice for female urologists and general surgeons. However, efforts should be made to reduce gender stereotypes in medicine, to ensure that patients receive the best care regardless of the sex of the surgeons.
